# Predicting Kidney Injury Following Valvular Heart Surgery in An Elderly Cohort: The Missing Links…

**DOI:** 10.21470/1678-9741-2021-0075

**Published:** 2022

**Authors:** Rohan Magoon

**Affiliations:** 1 Department of Cardiac Anaesthesia, Atal Bihari Vajpayee Institute of Medical Sciences (ABVIMS) and Dr. Ram Manohar Lohia Hospital, Baba Kharak Singh Marg, New Delhi, India. E-mail: rohanmagoon21@gmail.com

Dear Editor, I read with great interest the Carrascal et al.^[1]^ study centralizing the much-required clinical focus on the risk-factor modulation of a peculiarly multi-factorial cardiac surgery-associated acute kidney injury (CS-AKI). Despite a prospective consecutive evaluation of a large homogenous cohort (elderly patients undergoing valvular heart surgery) in the index study, certain points require authors’ comments and clarification to enhance the lucidity of their findings.

- While preoperative diabetes mellitus did not emerge as a baseline risk-predictor of CS-AKI (19.7% and 15.6% incidence in AKI and non-AKI population, respectively), the lack of elucidation of perioperative glucose homeostasis in the present study captivates attention, particularly when poor glucose control has been linked to an enhanced propensity for CS-AKI owing to an augmented oxidative stress, endothelial dysfunction and systemic inflammatory response^[2-4]^. Moreover, the authors also fail to delineate their institutional perioperative glucose management protocol.
- Talking of the hemodynamic predisposition to CS-AKI, there are additional caveats that merit elaboration. It is noteworthy that systemic venous hypertension has been demonstrated to be associated with poor renal outcomes in cardiac surgical patients manifesting as a congestive renal failure, as an elevated central venous pressure (CVP) is potentially transmitted backwards, reflecting as an accentuated renal venous pressure (RVP) which reduces effective renal perfusion pressure (mean arterial pressure-RVP), accelerating AKI progression. The aforementioned is heralded by the results of the study by Palomba et al.^[5]^ aimed at developing the ‘Acute Kidney Injury in Cardiac Surgery’ (AKICS) score, which adequately outline the superiority of postoperative CVP and low cardiac output as independent risk factors in predicting CS-AKI over other hemodynamic variables. Across the cardiac surgical population, the risk of AKI doubles once the postoperative CVP reaches a threshold value of 14 mmHg^[5]^. Ahead of the fact that estimated CVP at 6 h postoperatively has been found to be significantly associated with CS-AKI in the non-congested cardiac surgical subset^[6]^, possible links should definitely not be overlooked in valvular heart disease patients included in the present study who can be particularly prone to postoperative systemic venous congestion^[7]^.
- Lastly, the lack of comparative account for inotropes-vasopressors in the AKI and non-AKI groups or a standardized inotrope-vasopressor study protocol requires consideration while interpreting the findings.
